# Production and Characterization of Novel Recombinant Adeno-Associated Virus Replicative-Form Genomes: A Eukaryotic Source of DNA for Gene Transfer

**DOI:** 10.1371/journal.pone.0069879

**Published:** 2013-08-01

**Authors:** Lina Li, Emilios K. Dimitriadis, Yu Yang, Juan Li, Zhenhua Yuan, Chunping Qiao, Cyriaque Beley, Richard H. Smith, Luis Garcia, Robert M. Kotin

**Affiliations:** 1 Laboratory of Molecular Virology and Gene Therapy, National Heart, Lung and Blood Institute, National Institutes of Health, Bethesda, Maryland, United States of America; 2 National Institute of Biomedical Imaging and Bioengineering, National Institutes of Health, Bethesda, Maryland, United States of America; 3 School of Pharmacy, University of North Carolina at Chapel Hill, Chapel Hill, North Carolina, United States of America; 4 Biotherapies of Neuromuscular Diseases, Faculty of Health Sciences, University of Versailles, Saint-Quentin-en-Yvelines, France; University of Kansas Medical Center, United States of America

## Abstract

Conventional non-viral gene transfer uses bacterial plasmid DNA containing antibiotic resistance genes, *cis*-acting bacterial sequence elements, and prokaryotic methylation patterns that may adversely affect transgene expression and vector stability *in vivo*. Here, we describe novel replicative forms of a eukaryotic vector DNA that consist solely of an expression cassette flanked by adeno-associated virus (AAV) inverted terminal repeats. Extensive structural analyses revealed that this AAV-derived vector DNA consists of linear, duplex molecules with covalently closed ends (termed closed-ended, linear duplex, or “CELiD”, DNA). CELiD vectors, produced in Sf9 insect cells, require AAV *rep* gene expression for amplification. Amounts of CELiD DNA produced from insect cell lines stably transfected with an ITR-flanked transgene exceeded 60 mg per 5×10^9^ Sf9 cells, and 1–15 mg from a comparable number of parental Sf9 cells in which the transgene was introduced via recombinant baculovirus infection. In mice, systemically delivered CELiD DNA resulted in long-term, stable transgene expression in the liver. CELiD vectors represent a novel eukaryotic alternative to bacterial plasmid DNA.

## Introduction

Non-viral gene transfer typically uses bacterial plasmids to introduce foreign DNA into recipient cells. In addition to the transgene of interest, such DNAs routinely contain extraneous sequence elements needed for selection and amplification of the plasmid DNA (pDNA) in bacteria, such as antibiotic resistance genes and a prokaryotic origin of replication. Also, bacterial DNA preparations often contain endotoxin that can reduce gene transfer efficiency [1]. Ideally, DNA for non-viral gene transfer would contain only the gene of interest, be devoid of prokaryotic modifications that can trigger an innate immune response [1], [2], [3], [4], be in an exonuclease-resistant form, and lack detectable endotoxin contamination. Transgene persistence is also highly desirable for many gene therapy applications. We have observed that transgenes flanked by inverted terminal repeats (ITRs) from adeno-associated virus type 2 (AAV) can be amplified to high copy number in *Spodoptera frugiperda* (Sf9) cells in the presence of a recombinant baculovirus expressing the AAV replication proteins, Rep78 and Rep52. DNA produced in this manner consists solely of a transgene of interest with flanking AAV ITRs, and, since it is produced in eukaryotic cells, is devoid of prokaryotic DNA modifications and bacterial endotoxin contamination. Structural characterization revealed the ITR-flanked transgenes as exonuclease-resistant, linear DNA molecules with covalently closed ends. We have termed these closed-ended, linear duplex molecules “CELiD” DNA.

CELiD DNA is produced in Sf9 cells upon co-infection with two separate baculovirus expression vectors (BEVs): the first BEV bearing an ITR-flanked transgene, and a second BEV (designated Bac-Rep) encoding AAV non-structural proteins (Rep78 and Rep52) essential for ITR-mediated DNA replication. Alternatively, CELiD DNA can be rescued and amplified upon Bac-Rep infection of clonal Sf9 cell lines bearing a stably integrated rAAV vector genome. The structure of replicated ITR-flanked transgene DNA was analyzed using native and denaturing agarose gel electrophoresis, restriction endonuclease size mapping, atomic force microscopy (AFM), and exonuclease sensitivity assays. The results of these orthogonal assays established that the predominant structure of the replicated vector DNA is closed-ended, linear duplex monomers and dimers.

## Materials and Methods

### Ethics statement

The University of North Carolina at Chapel Hill Institutional Animal Care and Use Committee (IACUC) approved the animal study protocols.

### Sf9 insect cell culture

The *Spodoptera frugiperda* Sf9 cell line was obtained from Life Technologies, Corp. (Grand Island, NY, USA). Sf9 cells were grown in suspension in serum-free media (HyQ® SFX, HyClone, Logan, UT, USA). Cultures were maintained within vented-cap, polycarbonate Erlenmeyer flasks (Corning, Inc., Corning, NY, USA) in an orbital platform shaker (135 rpm agitation) at 27°C in ambient atmosphere. Cell density, size and viability were assessed using an automated cell counter (Cellometer, Nexcelom Bioscience, Lawrence, MA, USA) or flow cytometry (Guava EasyCyte Mini, Millipore Corp., Billerica, MA, USA).

### Plasmid pFBGR-bsd construction

The blasticidin-S deaminase (bsd) gene (along with the *Orgyia pseudotsugata* immediate early-1 (Op IE-1) and EM7 promoters) was PCR-amplified from pIB/V5-His/CAT (Life Technologies, Corp.) using the following primer pair: 5′-ATAAGCTTACGCTCAGTGGAACGAAAAC-3′ and 5′-ATAAGCTTGACGTGTCAGTGTCAGTCCTGCTCCT-3′. The 865 bp PCR product was digested with HindIII (sites underlined in primer sequence) and ligated into HindIII-digested pFBGR (5).

### Establishing stable Sf9/ITR-GFP cell lines for high-yield CELiD-GFP DNA production

Sf9 cells were transfected with pFBGR-bsd using Cellfectin Transfection Reagent (Life Technologies, Corp.). At three days post-transfection, antibiotic-resistant cells were selected by the addition of blasticidin-S HCl (50 µg/ml) (Life Technologies, Corp.) to the growth medium. After two weeks in selective medium, blasticidin-resistant (bsd^r^) clones were derived by single-cell dilution or direct colony transfer techniques. The bsd^r^ clones were expanded in insect cell culture medium supplemented with 10% fetal bovine serum (FBS) (HyClone) and blasticidin-S HCl (10 µg/ml) for 2 to 3 additional passages, then returned to serum-free medium with 10 µg/ml blasticidin-S HCl. After an additional 12 passages, blasticidin-S HCl was omitted from the medium and the cell lines were expanded for analysis.

For functional screening, clonal Sf9/ITR-GFP cell lines were infected (MOI  = 5) with a recombinant baculovirus, Bac-Rep, expressing the AAV type 2 Rep78 and Rep52 proteins [5] and analyzed for induced GFP expression. Clonal Sf9/ITR-GFP cells with the highest levels of GFP fluorescence were expanded for CELiD-GFP DNA preparation.

### Production and purification of CELiD-GFP DNA from a clonal Sf9/ITR-GFP cell line

Clonal Sf9/ITR-GFP cells were seeded at 2×10^6^ cells/mL and infected with Bac-Rep (MOI  = 1 to 3). Cell viability and diameter were monitored daily until the cell diameter increased to 18–20 µm (uninfected cell diameter 14–15 µm), indicating that the cells were in the late stages of the viral infection. Extrachromosomal DNA was extracted from the Bac-Rep-infected, Sf9/ITR-GFP cells using a commercially available plasmid isolation kit (Qiagen, Valencia, CA, USA). CELiD production was monitored by agarose gel electrophoresis and ethidium bromide staining of extrachromosomal DNA. The presence of a 2.7 kb band after Bac-Rep infection indicated that the “proviral” rAAV-GFP genome was rescued from chromosomal DNA and amplified as an episomal element.

### CELiD DNA production in parental Sf9 cells

CELiD DNA was produced in parental Sf9 cells by co-infection with two separate baculovirus expression vectors (BEV): Bac-Rep and a second BEV bearing an ITR-flanked transgene, such as Bac-GFP or Bac-LacZnls. Infected *S*f9 cells were harvested once the mean cell diameter increased by 4–5 µm and the percent viability decreased to 80–90%. CELiD DNA was isolated using a commercially available plasmid purification kit (Qiagen).

### Construction of pFB-TBG-GFP and baculovirus production

A thyroxine-binding globulin promoter (TBG)-GFP cassette (1797 bp) was isolated from pENN AAV TBG PI-eGFP (kindly provided by Dr. Charles P. Venditti, NHGRI, NIH) following digestion with NheI and PspXI. The ca. 1800 bp TBG-eGFP fragment was transferred into the backbone of pFBGR after the GFP transcription cassette was removed from pFBGR by digestion with SpeI and XhoI (retaining the AAV2 ITRs and transposase Tn7 recombination signals). The resulting pFB-TBG-GFP (7074 bp) plasmid consists of the AAV2 ITRs flanking the TBG promoter and GFP open reading frame. Recombinant baculovirus stocks were generated using the Bac-to-Bac System (Life Technologies, Corp.) with modifications to the plasmids previously described [5]. Briefly, competent *E. coli* DH10Bac cells were transformed with pFB-TBG-GFP. Following selection on triple antibiotic agar plates (containing 50 µg/ml kanamycin, 7 µg/ml gentamicin, 10 µg/ml tetracycline, and X-gal 100 µg/ml), several white colonies were picked and expanded in small volume cultures. Bacmid DNA was extracted and used to transfect Sf9 cells. Three days after transfection, baculovirus-containing supernatant (passage 1 or “P1”) was expanded two more rounds by adding infected-cell supernatant to fresh Sf9 cells (1∶100; v:v). Infected cells were cryopreserved at P3, and served as a source of infectious baculovirus as previously described [6].

### Construction of pFB-CMV-LacZnls and baculovirus production

Plasmid pFB-CMV-LacZnls was produced by isolating the 4.1 kb LacZnls gene (encoding a nuclear-localized β-galactosidase protein) from XbaI- and SalI-digested pAAV2.1-CM (pCMV-LacZnls), kindly provided by Dr. Xiao Xiao (UNC-Chapel Hill, Chapel Hill, NC), and ligating this fragment to the 5.2 kb fragment of SpeI- and XhoI-digested pFBGR, thus replacing the GFP reporter gene with LacZnls coding sequences. Competent DH10Bac cells (Life Technologies, Corp.) were transformed with pFB-CMV-LacZnls to yield Bac-LacZnls bacmid DNA. Recombinant Bac-LacZnls bacmid isolates were identified by PCR screening using M13/pUC reverse sequencing primer (5′-AGCGGATAACAATTTCACACAGG-3′) and a synthetic LacZ insert primer (5′-CGAGAAGTACTAGAGGATCA-3′). Bacmid DNA was transfected into Sf9 cells to yield an infectious baculovirus, Bac-LacZnls, which was further amplified by serial passage in Sf9 cells.

### Denaturing agarose gel electrophoresis

Alkaline (denaturing) agarose gels were prepared by dissolving agarose (SeaKem LE agarose, BioWhittaker Molecular Applications, Rockland, ME, USA) in boiling water and cooling to near gelling temperature. Alkaline gel solution concentrate (10x) was added to a 1x final concentration just before the gels were poured. The 1x alkaline gel running buffer and gel solution composition is: 50 mM NaOH, 1 mM EDTA. Using 10x concentrate, DNA samples were adjusted to 1x alkaline gel solution conditions and supplemented with 2.5% Ficoll-400 and 0.05% bromophenol blue. Electrophoresis was performed at constant voltage (30–60 V) for 3–6 hr.

### Time-course analysis and requirements for CELiD rescue and amplification in Sf9 cells

Clonal Sf9/ITR-GFP cells were inoculated with various amounts of Bac-Rep stock. Periodically, cells were harvested and extrachromosomal DNA was recovered using a commercially available DNA isolation kit (Qiagen Plasmid Mini Kit). Extracted DNA was examined by either agarose gel electrophoresis or by PCR with GFP-specific primer pairs for quantitative determination of CELiD DNA amounts.

For western blotting, cell proteins were fractionated by SDS-polyacrylamide gel electrophoresis and transferred to nitrocellulose membranes. The membranes were incubated in blocking buffer (BB) composed of 5% non-fat dry milk (w:v) in phosphate-buffered saline plus 0.05% Tween-20 (PBST) for 1 hr at ambient temperature with orbital agitation. After washing the membranes in wash buffer (WB) composed of 3% non-fat dry milk in PBST, membranes were incubated with the appropriate primary antibody solution (diluted in BB) either at ambient temperature (1 hr) or 4°C (overnight) with continuous orbital agitation. The following primary antibodies and dilution ratios were used: 1. anti-AAV Rep mouse monoclonal antibody (mAb) 303.9 (American Research Products, Inc., Waltham, MA, USA), 1∶200 dilution. 2. anti-baculovirus envelope glycoprotein gp64 mouse mAb (eBioscience San Diego, CA, USA), 1∶1000 dilution. 3. anti-GAPDH mAb (Ambion, Life Technologies, Corp.), 1∶5000 dilution. 4. HRP-conjugated anti-GFP mAb (Fitzgerald Industries International, Acton, MA, USA), 1∶5000 dilution. After incubation, primary antibody solutions were removed and membranes were washed in WB (3×5 mins). Non-conjugated mAbs were incubated with secondary antibody solution (goat, anti-mouse horseradish peroxidase (HRP)-conjugate (1∶5000) (Sigma, St. Louis, MO, USA) for 1 hr, and then washed with WB as above. HRP activity was detected by enhanced chemiluminescence (ECL) (SuperSignal West Dura chemiluminescent substrate, Thermo Scientific, Rockford, IL, USA). Images were acquired using a digital gel documentation instrument (G:Box Chemi, Syngene USA, Frederick, MD, USA).

### Atomic force microscopy (AFM) imaging of CELiD–GFP DNA

The 2.7 kb monomer and 5.4 kb dimer CELiD–GFP replicative-form DNAs were purified from gel slices following agarose gel electrophoresis (using QIAEX II gel extraction kit, Qiagen) and dissolved in molecular biology grade H_2_O (Quality Biological, Inc., Gaithersburg, MD, USA).

Prior to applying the DNA samples, AFM mica substrates were modified with aminopropylsilatrane (APS). APS solution (∼150 nM in 50 µl) was applied to freshly cleaved mica disks (12 mm diameter) for 30 minutes at ∼20°C. The disks were then rinsed thoroughly with dH_2_O and dried in a nitrogen stream. DNA solutions of appropriate concentrations (typically, on the order of 1 µM base-pair concentration) were applied to the APS-treated mica and incubated approximately 10 min at room temperature. The samples were then gently rinsed with dH_2_O and dried in an argon stream before imaging.

The effects of different solvents on the topological features of the sample DNA were examined by AFM using: 1. Buffered saline (500 mM NaCl and 20 mM HEPES, pH 7.4), 2. 50% formamide (sample heated at 60°C for 30 minutes to denature the DNA), and 3. Water (dH_2_O).

Samples were imaged with an AFM instrument (MultiMode-PicoForce AFM with Nanoscope V controller, Bruker Nano, Santa Barbara, CA, USA). Imaging was performed in oscillating mode (tapping) under ambient conditions using silicon cantilevers with nominal stiffness of ∼42 N/m and resonance frequencies in the 300 kHz range (OTESPA by Olympus or TESP-SS by Bruker Nano). Imaging data were preprocessed using Nanoscope v7.31. DNA lengths were measured using algorithms developed in conjunction with image processing software based on NIH ImageJ (http://rsb.info.nih.gov/ij/), Matlab (Mathworks, Natick, MA, USA) and Origin (OriginLab, Northampton, MA, USA).

The lengths of extended DNA chains with clearly discernible paths were determined by contour tracing. For overlapping and entangled chains, we developed new algorithms that made use of the volume information contained in the AFM topographic images. Volumes were measured by adding pixel heights using the Particle Analysis module of NIH ImageJ.

### CELiD DNA expression *in vitro*

Transfections were conducted using either GFP or nuclear-localized β-galactosidase (LacZnls) reporter cassettes. HEK 293 cells were seeded in 6-well tissue culture plates one day before transfection to achieve 90% confluence at the time of use. Transfections were performed using a commercially available liposome-based transfection reagent (Lipofectamine® 2000, Life Technologies, Corp., Carlsbad, CA, USA) following the manufacturer's suggested protocol. For GFP expression assays, HEK 293 cells were transfected using 4 µl of lipid reagent per well and equivalent copy numbers of reporter DNA molecules: CELiD–GFP DNA (1.48 µg per well) or plasmid-GFP DNA, pFBGR (4 µg per well). At day 6 post-transfection, cells were examined for GFP expression by fluorescence microscopy.

For LacZ expression assays, HEK 293 cells were seeded in 6-well tissue culture plates and transfected with equal weight amounts of CELiD-LacZnls (2 µg) or pFB-CMV-LacZnls (2 µg) using 3 µl of lipid reagent per well. Three days post-transfection, β-galactosidase (β-gal) activity was detected by staining with the chromogenic substrate, X-gal. Briefly, cells were rinsed with PBS, and then fixed in 2% formaldehyde, 0.1% glutaraldehyde in PBS for 2 minutes. After washing the cells three times with PBS, freshly made X-gal staining buffer (1 mg/ml X-gal, 2 mM MgCl2, 5 mM potassium ferrocyanide, and 5 mM potassium ferricyanide in PBS, warmed to 50°C) was added, and the cells were incubated at 37°C for 30 min to 24 hr in the dark. After incubation, samples were washed three times (each for 5 min) in room temperature PBS, and examined by light microscopy.

### Hydrodynamic injection of DNA and *in vivo* gene expression detection

Outbred male ICR mice at 2 months of age were used for hydrodynamic DNA injection via the tail vein according to the method described by Liu et al. [7]. Each mouse was injected with 1.7 ml of saline containing circular plasmid DNA or CELiD DNA. For β-galactosidase activity, 10 µg of pCMV-LacZnls or 1 µg of CELiD-LacZnls DNA was used. Mice were sacrificed periodically (1, 3, and 7 days, as well as 5 weeks, post-injection) and liver tissue collected and snap-frozen in liquid nitrogen for X-gal staining of tissues. The liver tissues were cryo-sectioned (15 µm thickness), fixed and X-gal stained according to a standard method [8]. Samples were counter-stained with eosin, dehydrated with ethanol, and mounted for imaging.

For TBG-GFP gene expression *in vivo*, 10 µg of either CELiD-TBG-GFP or circular plasmid pTBG-GFP in 1.7 ml saline was injected via the tail vein. Mice were sacrificed at 1, 3, and 10 wks post-injection, and livers samples were processed as above. GFP expression within the liver sections was detected by fluorescence microscopy. N = 3 to 4 animals per experimental time point.

### *In vivo* DNA copy number determination

DNA was extracted from transfected liver tissue using the DNeasy Blood and Tissue kit (Qiagen). Vector copy number was determined by quantitative PCR (qPCR). TaqMan assays of the endogenous mouse glucagon gene were used to normalize vector copy numbers. The sequences of the mouse glucagon gene primers and probe used were: Glucagon-real-F (mouse), 5′-AAGGGACCTTTACCAGTGATG TG-3′; Glucagon-real-R (mouse), 5′-ACTTACTCTCGCCTTCCTCGG-3′; TaqMan mouse glucagon probe, 5′-FAM-CAGCAAAGGAATTCA-MGB-3′. The CELiD-LacZnls-specific PCR primers and probe were: CMV-forward, 5′-GTATGTTCCCATAGTAACGCCAATAG-3′; CMV-reverse, 5′-GGCGTACTTGGCATATGATACACT-3′; CMV-probe, 5′-FAM-TCAATGGGTGGAGTATTTA-3′. The following primer pair was used to detect TBG-GFP: forward, 5′-GGAAAGTCCCTATTGACGTT-3′; reverse, 5′-GGAAAGTCCCTATTGACGTT-3′.

### Statistical analysis

Quantitative results are expressed as the mean plus or minus (±) the standard deviation (SD). Statistical significance analysis of the data was performed using an unpaired two-sample equal variance and two-tail distribution Student's t-test (t-test). A *p*-value of less than 0.05 was considered statistically significant as indicated by asterisks: * *p*<0.05, ** *p*<0.01, *** *p*<0.001.

## Results

### Characterization of replicative-form vector DNA produced in stably-transfected Sf9 cell lines

**Figure 1:**
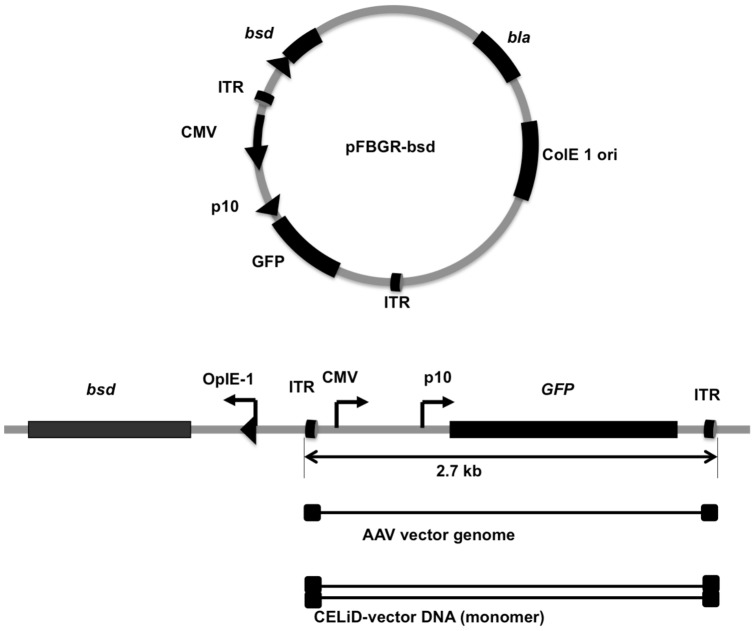
Schematic representation of plasmid DNA used for producing stable cell lines. Plasmid pFBGR-bsd contains the GFP gene under the dual control of the cytomegalovirus IE promoter (CMV) and baculovirus p10 promoter (p10) flanked by AAV-2 inverted terminal repeats (ITR). *bla*, β-lactamase (ampicillicin-resistance gene). *bsd*, blasticidin-S deaminase gene. ColE1, bacterial origin of replication. (Lower) Linear illustration of pFBGR-bsd indicates the rescued forms of the ITR-flanked transgene. The linear, single-stranded AAV virion genome is represented by a solid thin line flanked by the inverted terminal repeats (ITRs, filled rectangles). The duplex CELiD-vector DNA is represented by the open rectangle flanked by AAV ITRs.

**Figure 2:**
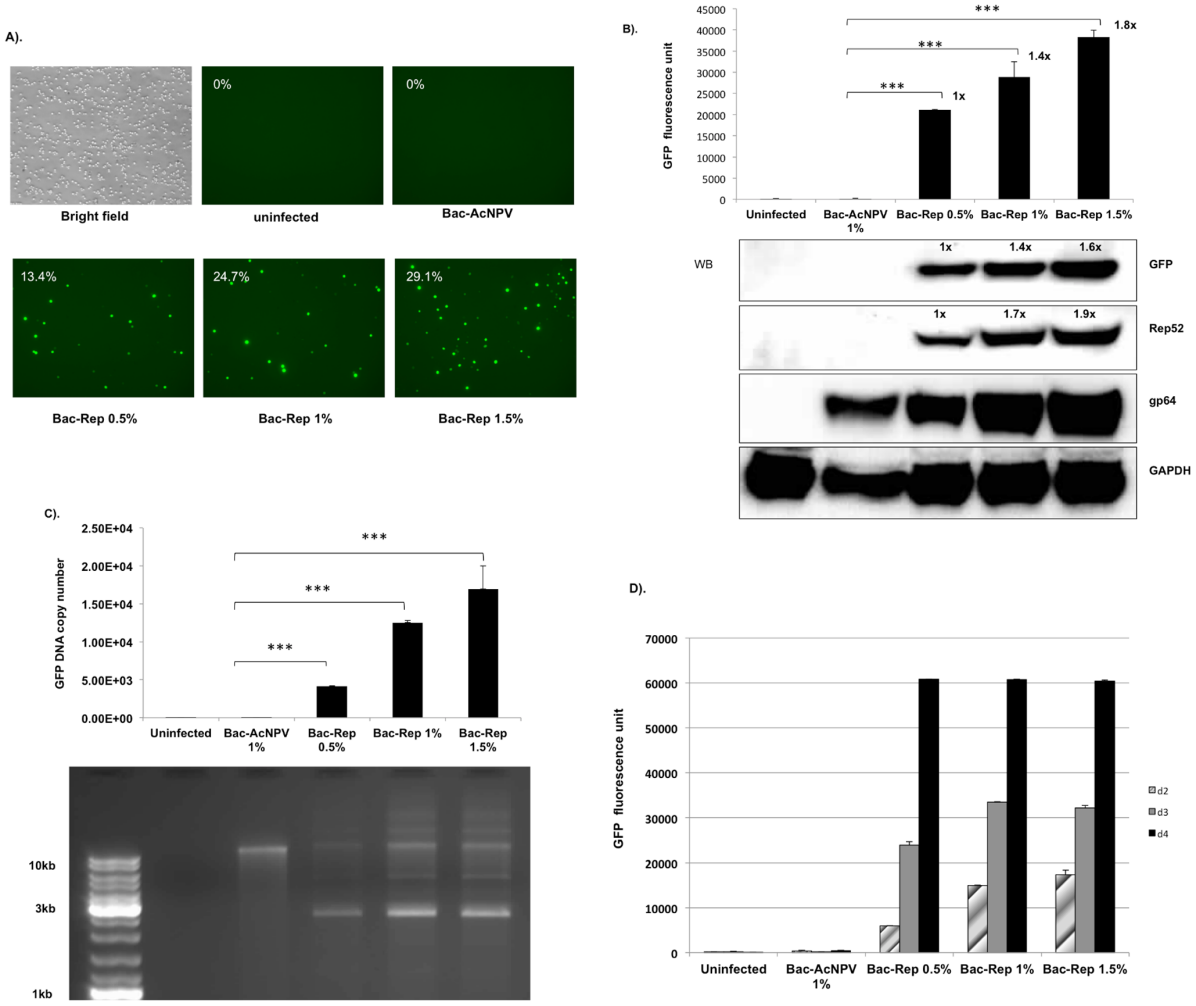
AAV Rep protein-dependent expression of GFP. (A) Induction of GFP expression in blasticidin-S resistant Sf9/ITR-GFP cells in response to Bac-Rep infection. The clonal Sf9/ITR-GFP cell line contains a stably integrated copy of pFBGR-bsd. Uninfected cells or cells infected with wild-type baculovirus (Bac-AcNPV) lack detectable GFP expression as determined by FACS analysis. (upper row, 0% GFP positive cells). Addition of Bac-Rep inoculum (0.5%, 1.0%, and 1.5%; v:v) resulted in a dose-dependent increase in the number of GFP-positive cells (lower row). Images were obtained 3 days after infection. Magnification, 10x objective used for all images. (B) Quantitative analysis of GFP induction as a function of Bac-Rep dose. Cells exposed to increasing doses of Bac-Rep were harvested on day 3 post-infection. Fluorescent emission intensities were assessed from equivalent amounts of cell lysates (80 µg protein), using the fluorescent reader function of a real-time thermocycler (excitation 450–490 nm; emission 515–530 nm) (upper panel), *** indicates t-test significance (p<0.001). The relative fold-increase in GFP fluorescence is indicated by the values next to each bar. Protein concentrations were determined in the lysates and approximately 120 µg of each sample was fractionated electrophoretically using SDS-polyacrylamide gels. Proteins were electroblotted onto nitrocellulose membranes for western blot detection of GFP, Rep52, gp64, and GAPDH (used as a protein loading and transfer standard, lower panel). Relative levels of protein are indicated by the values above the protein band. (C) Analysis of the increase of GFP-vector DNA in response to Bac-Rep infection. Low molecular weight DNA was recovered from the cells and the quantity of GFP vector genomes determined using real-time qPCR (upper panel). Both the uninfected cell control and the wild-type baculovirus-infected cell control lysates produced a relatively low PCR signal (150 and 175 copies per cell, respectively). The GFP vector DNA copy number increased to 21087 copies per cell with 0.5% (v:v) Bac-Rep. At 1% (v:v) Bac-Rep, the copy number increased to 28862 copies per cell and 1.5% (v:v) Bac-Rep increased the copy number to 38286 per cell. The statistical significance was determined by Student's t-test. ***, p<0.001. Lower panel – The samples were analyzed by agarose-ethidium bromide gel electrophoresis (1% agarose). No detectable CELiD DNA was recovered from uninfected cells. A band >10 kb appears in lysates from all baculovirus-infected cells. (D) Time-course of GFP fluorescence. Sf9/ITR-GFP cells were inoculated with either wtBac (Bac-ACNPV) or Bac-Rep (0.5%, 1%, and 1.5% (v:v)). GFP fluorescence was measured for the indicated cellular lysate at 1, 2, and 3 days post-infection.

A plasmid (pFBGR-bsd) containing a blasticidin-S deaminase selectable marker linked to an AAV ITR-flanked green fluorescent protein (GFP) reporter gene under the regulatory control of the CMV IE promoter and the baculovirus p10 promoter is shown in Fig. 1. Sf9 cell lines were produced by transfection with pFBGR-bsd and selection for stable blasticidin-S deaminase expression. Infection of blasticidin-resistant cells with a recombinant baculovirus expressing the AAV2 *rep* gene (Bac-Rep) induced GFP expression and amplification of the AAV ITR-flanked GFP transgene in a dose dependent manner (Fig. 2). In contrast, infection with wild-type baculovirus (Bac-AcNPV) had no effect on GFP expression. Characterization of the relative amounts of the Rep and GFP proteins by western blot analysis combined with quantification of GFP-associated fluorescent intensity indicated a direct correlation between Rep protein expression and the induction of GFP activity in infected cells (Fig. 2B).

To test the hypothesis that Rep protein expression results in rescue and amplification of the integrated GFP vector genome, extrachromosomal DNA was extracted from stably transfected Sf9/ITR-GFP cells and analyzed using quantitative PCR (qPCR) (Fig. 2C, upper panel) and agarose gel electrophoresis (Fig. 2C, lower panel). A unit-length 2.7 kb band is apparent in the Bac-Rep-infected cells, but not in cells infected with wild-type (wt) baculovirus (Bac-AcNPV). Using qPCR, it was determined that the copy number of the GFP target sequence increased to greater than 15,000 copies per cell, in proportion to the amount of Bac-Rep added to the culture (Fig. 2C, upper panel).

**Figure 3:**
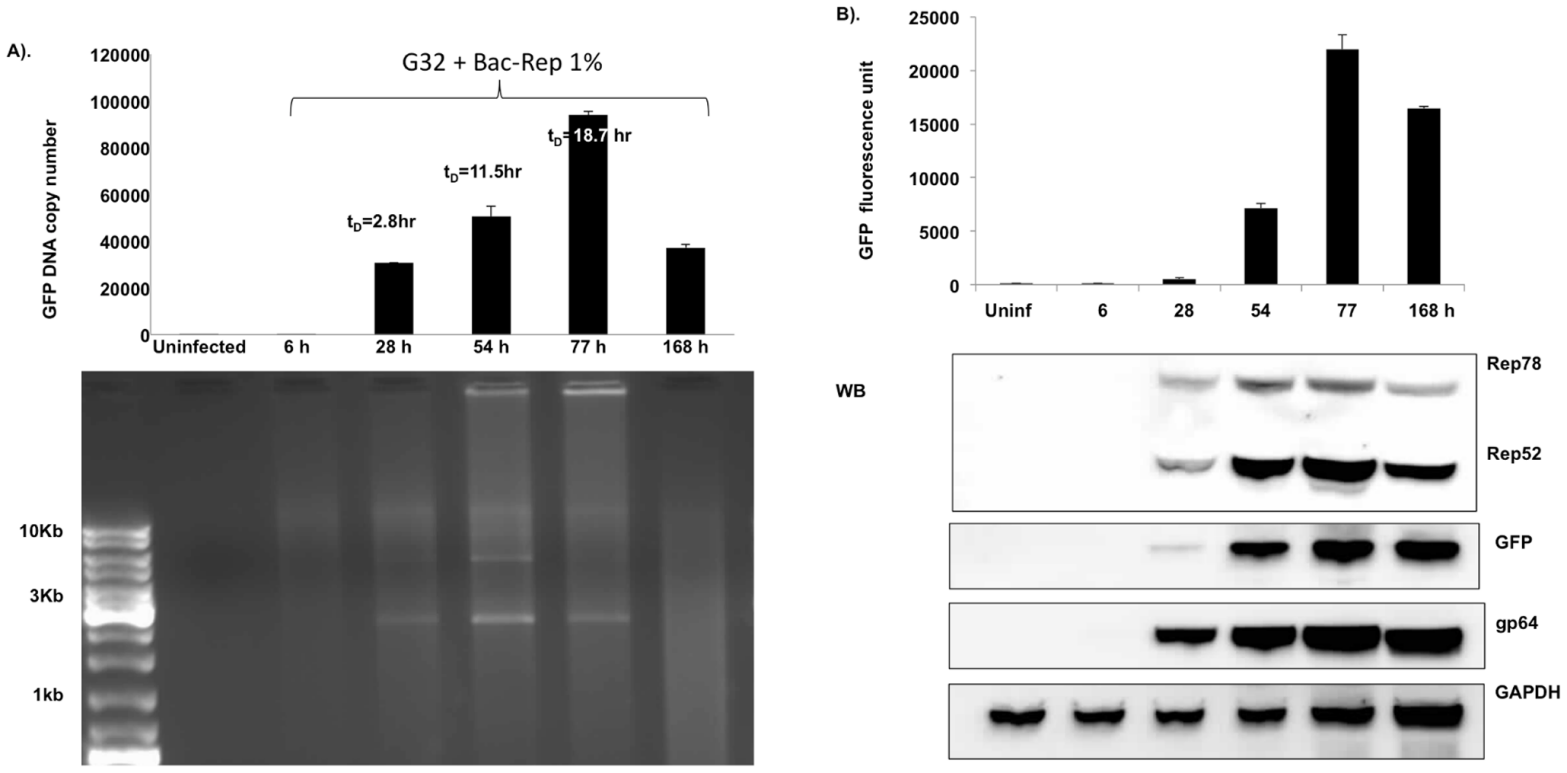
Rate of GFP vector DNA accumulation. (A). Cells were inoculated with 1% (v:v) Bac-Rep stock and sampled at 6, 28, 54, 77, and 168 hr post-infection. The GFP-vector DNA content was determined by qPCR (upper panel). The doubling time of the GFP vector DNA was determined using the following algorithm: t_D_  =  (t_2_–t_1_) log 2/log (t_2_/t_1_), where t_D_ is the doubling time and t_n_ is the GFP copy number at a given time point. The t_D_ (28 hr)  = 2.8 hr, t_D_ (54 hr)  = 11.5 hr, and t_D_ (77 hr)  = 18.7 hr. (B) Time course of protein expression in Sf9/ITR-GFP cells. GFP fluorescence was measured in cell lysates obtained from the time-course described in (A) (upper panel). Aliquots from each time point were fractionated on SDS-polyacrylamide gels and transferred to nitrocellulose membranes for western blot analysis to detect Rep proteins, GFP proteins, gp64, and GAPDH (used as a loading and transfer control).

During a time-course experiment, higher doses of Bac-Rep led to a proportional increase of GFP fluorescence observed on both day 2 and day 3 post-infection. By day four, the fluorescence intensity reached a maximum value regardless of the initial multiplicity of infection (Fig. 2D). The rate of accumulation of the GFP vector DNA was analyzed using samples of Sf9/ITR-GFP cells infected with 1% (v:v) of baculovirus-containing supernatant (Fig. 3A). The GFP vector genome copy number increased non-linearly over three days. At 168 hr post-infection, the amount of GFP vector genomes decreased, presumably due to loss of cell viability. The staining intensities of the monomer and dimer replicative-form DNAs (2.7 and 5.4 kb, respectively) (Fig. 3A, lower panel) indicate that the peak level of amplification occurred at the 55 hr time point. The rate of Rep 78 and Rep 52 expression reached a maximum at 77 hr post-infection, then declined at 168 hr (17% of 77 hr Rep expression) (Fig. 3B). The amount of GFP detected by western blot decreased slightly from 77 to 168 hr (69% of 77 hr GFP expression) (Fig. 3B).

### Characterization of CELiD DNA

**Figure 4:**
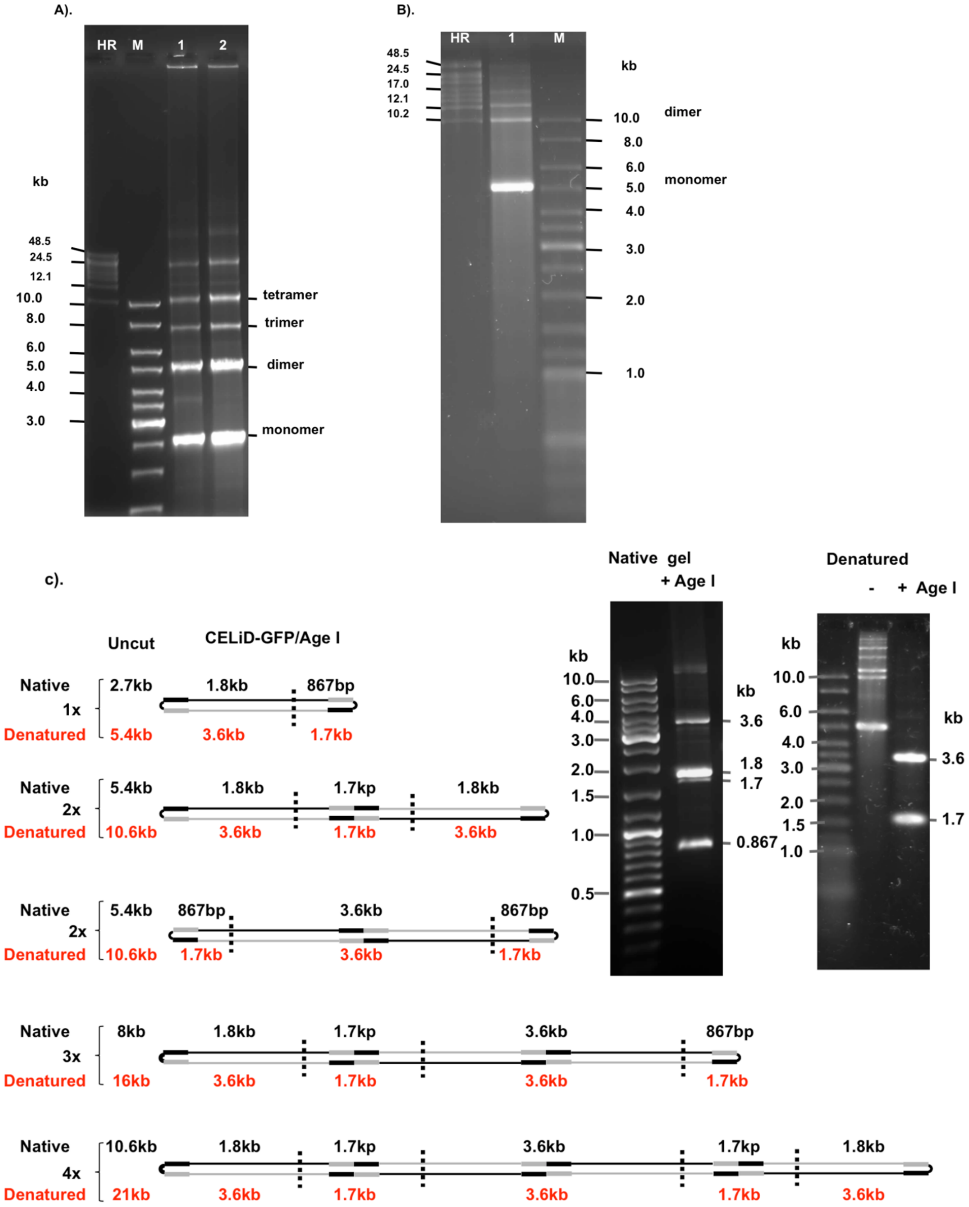
Native and denaturing agarose gel electrophoresis of CELiD-GFP DNA. (A) Native agarose gel electrophoresis (0.4% agarose, 1x TAE buffer). CELiD DNA resolved as a 2.7 kb monomer and associated multimeric concatomers. Lane 1: CELiD-GFP DNA produced from co-infecting parental Sf9 cells with Bac-Rep and a baculovirus bearing an AAV ITR-flanked GFP cassette, Bac-GFP. Lane 2: CELiD-GFP DNA produced from an Sf9/ITR-GFP cell line bearing a stably integrated AAV GFP vector genome. M: 1 kb DNA ladder. HR: high-range DNA ladder. The positions of various replicative-form CELiD DNAs are indicated: monomer, dimer, trimer, and tetramer. (B) Denaturing agarose gel electrophoresis (0.7% alkaline agarose). CELiD-GFP DNA conformers appear predominantly as a 5.4 kb band, with dimer and higher order forms detectable. (C) Restriction map. Restriction endonuclease AgeI has one recognition site in the CELiD monomer, generating two fragments of 1801 bp and 867 bp, respectively. Schematic representation of the AgeI recognition site(s) in the monomer (1x), dimer (2x) and multimer (3x and 4x) forms of CELiD-GFP DNA. CELiD-GFP monomer: 2668 bp. The black rectangles indicate the positions of the AAV 5′ ITRs, and the gray rectangles indicate the positions of the AAV 3′ ITRs. The two dimer figures represent tail-to-tail and head-to-head configurations. The predicted DNA length and fragments are indicated. Top right, images of native (1% agarose, TBE buffer) and denaturing (0.7% agarose, alkaline buffer) agarose gel electrophoresis of AgeI-digested CELiD-GFP DNA. “−” indicates uncut CELiD-GFP DNA, “+” indicates AgeI-digested DNA.

Extrachromosomal DNA was extracted from Bac-Rep-infected Sf9/ITR-GFP cells and resolved on either native or denaturing agarose gels. Under native conditions, the extrachromosomal DNA appeared predominantly as monomeric (2.7 kb) and dimeric (5.4 kb) forms of the vector genome, within a ladder of vector genome multimers (Fig. 4A). Under denaturing conditions, however, the extrachromosomal DNA presented predominantly as approximately 5.4 kb and 10.8 kb bands (Fig. 4B). The change in mobility under denaturing conditions suggested that the monomeric and dimeric vector genomes are duplex molecules with at least one covalently closed end.

To determine whether the ends of the linear ITR-flanked GFP transgene are covalently closed, extrachromosomal DNA recovered from Sf9/ITR-GFP cells was treated with the restriction endonuclease AgeI and resolved using either native or denaturing agarose gel electrophoresis (Fig. 4C) (see also Fig. S1). Following digestion with AgeI, the extrachromosomal DNA resolved as two major bands, of 1.8 kb and 0.9 kb, and two minor bands of 3.6 kb and 1.7 kb (Fig. 4C, right). The 1.8 kb and 0.9 kb bands are as expected for digestion of a vector genome monomer with AgeI, whereas the 3.6 kb and 1.7 kb bands are consistent with head-to-head and tail-to-tail concatamers of multimeric vector genomes (Fig. 4C, left). Under denaturing conditions, the AgeI digestion products appeared as just two bands (of 3.6 kb and 1.7 kb). Lack of the 1.8 kb and 0.9 kb bands suggests that the replicated vector genomes are covalently closed at both ends of the DNA duplex. Use of other restriction endonucleases produced results consistent with this model (Fig. S1).

**Figure 5:**
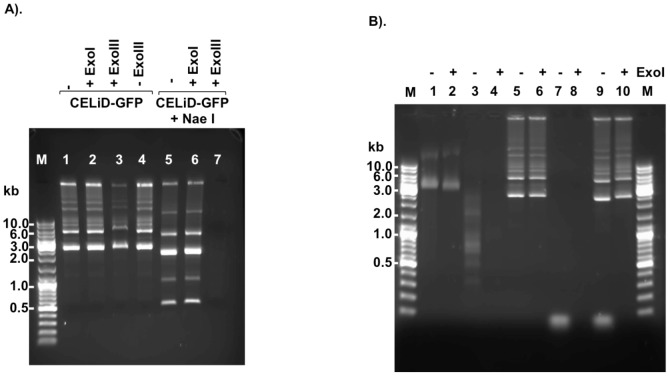
CELiD-GFP DNA sensitivity to exonuclease I and exonuclease III. *E. coli* exonuclease III (ExoIII) removes nucleotides processively (3′ –>5′) from DNA initiating at a 3′-OH of either blunt-ended or 5′ protruding duplex DNA. *E. coli* exonuclease I (ExoI) degrades single-stranded DNA processively in a 3′ to 5′ direction. (A) Total CELiD DNA (1 µg) was incubated with either ExoI (20 units) or ExoIII (100 units), either without prior restriction enzyme (RE) digestion (left) or following RE digestion (right). Lane M: DNA size ladder. Lane 1: untreated CELiD DNA. Lane 2: CELiD DNA treated with ExoI. Lane 3: CELiD DNA treated with ExoIII. Lane 4: untreated CELiD DNA. Samples in lanes 5, 6, 7 were digested with NaeI prior to exonuclease treatment. Lane 5: no exonuclease control. Lane 6: ExoI treatment. Lane7: ExoIII treatment. (B) Additional substrates as controls for ExoI activity. The φX174 virus genome is a 5386 nt, single-stranded, closed circular DNA molecule. ExoI treatment is indicated above each lane by a “+” or “–” sign. Lanes 1 and 2: φX174 DNA. Lanes 3 and 4: φX174 DNA digested with HaeIII. Lanes 5 and 6: CELiD DNA. Lanes 7 and 8: single-stranded synthetic oligonucleotide (25mer). Lanes 9 and 10: Mixed CELiD DNA and 25mer.

As second indication of whether the ends of the vector DNA are covalently closed, isolated extrachromosomal DNA was subjected to digestion with either *E. coli* exonuclease I (ExoI) or exonuclease III (ExoIII). ExoI degrades single-stranded DNA in the 3′ to 5′ direction [9]. ExoIII progressively degrades duplex DNA in a 3′ to 5′ direction from 3′-recessed or blunt-ended DNA molecules (substrates with 3′ protruding ends greater 4 nt are resistant) [10], [11]. Figure 5 shows that CELiD DNA and circular single-stranded φX174 DNA were resistant to ExoI digestion, while a single-stranded, synthetic oligonucleotide or endonuclease HaeIII-digested φX174 DNA were susceptible. As shown in Fig. 5A, native CELiD DNA was resistant to ExoIII digestion; however, after treatment with restriction enzyme NaeI, the vector DNA became susceptible to ExoIII activity. Together with the denaturing agarose gel electrophoresis results, these observations are consistent with a replicative-form DNA bearing a closed-ended DNA duplex structure.

### Atomic Force Microscopy of CELiD – vector DNA

**Figure 6:**
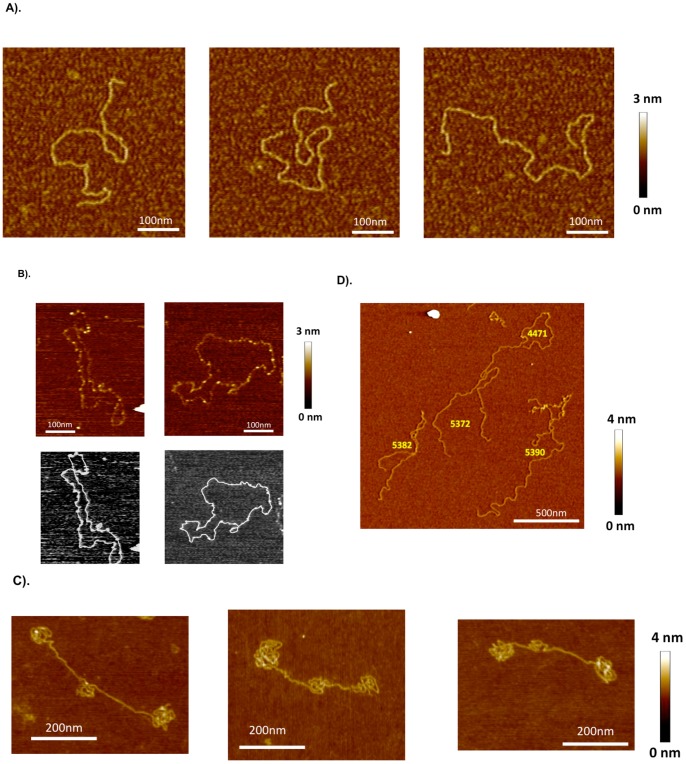
AFM images of CELiD vector DNA chains adsorbed onto APS-treated mica. (A) Typical images of the CELiD-GFP monomer deposited from aqueous solution (dH_2_O). The monomer is indistinguishable from standard double-stranded, linear DNA. All images are 450×450 nm. (B) CELiD-GFP monomers adsorbed onto mica substrate immediately after being exposed to denaturing conditions. The height of the chain is about half that of the native monomer chain confirming that the loops are single-stranded. These monomers are closed loops with randomly located, condensed regions (brighter spots along the loops). The lengths of these loops are consistent with denatured monomers supporting the model of a covalently closed, duplex conformation under native conditions. Top images are of the denatured monomers. Bottom images show the corresponding traced loops. (C) Images of individual CELiD-GFP dimers adsorbed from H_2_O. The chains are double-stranded, linear DNA with lengths twice that of the monomer and exhibit a characteristic conformation with three condensed regions, one located centrally and the other two at opposite ends of the molecule. (D) Under high salt conditions (0.5 M NaCl), the condensed regions of the CELiD-GFP dimer relax and the chains take on conformations typical of double-stranded, linear DNA with length twice that of the monomer CELiD vector DNA. Numbers in yellow indicate the chain length in bp, estimated by path tracing.

Atomic force microscopy (AFM) was used to visualize purified CELiD DNA (Fig. 6). The 2.7 kb CELiD-GFP vector genome appeared as an extended, linear duplex molecule with an estimated mean contour length of 2701 (±607) base pairs (Fig. 6A). Results of alkaline agarose gel electrophoresis analysis predict that, upon denaturation, the 2.7 kb CELiD-GFP monomer should occur as an approximately 5400 base-long, single-stranded, circular molecule. Although maintaining CELiD DNA in a single-stranded state proved difficult, several predominantly single-stranded DNA molecules with closed contours were observed under denaturing conditions (Fig. 6B). The lengths of extended DNA chains with clearly discernible paths were determined by contour tracing and were consistent with a covalently closed, single-stranded monomer.

AFM analysis of the 5.4 kb CELiD-GFP DNA dimer revealed an interesting conformation. The termini of the dimer appear as compacted, tortuous structures with a similar, but less extensive, structure centrally located (Fig. 6C). The contours of these molecules could not be traced; however, the lengths were estimated using total molecular volumes. The calculated DNA lengths averaged 5480 bp (±310 bp; n = 23) in agreement with the predicted size of 5336 bp. The positions of the convoluted regions of the dimer DNA correspond to the locations of the AAV ITRs. The AAV2 Rep78 protein, required for CELiD-vector DNA production, has sequence specific DNA binding activity. Multimers of Rep78 have been shown to non-covalently interact with a motif repeated in the AAV ITR [12], [13]. Western blots of nuclease-treated CELiD-vector DNA preparations detected the presence of both Rep78 and Rep52 (Fig. S2). To investigate whether Rep proteins, or other proteins, binding to the DNA contributed to the convoluted structures, the ionicity was increased to potentially disrupt non-covalent protein-DNA interactions. In the presence of 0.5 molar NaCl, the 5.4 kb CELiD-GFP DNA transitioned to an extended conformation (Fig. 6D). The lengths of several such relaxed DNA molecules were measured by contour tracing (5337±118 bp; n = 18), and were consistent with the lengths estimated for the convoluted forms using the volume per unit length estimates. The measured lengths agree with the predicted size of the duplex dimer (5336 bp).

### CELiD – vector DNA expression *in vitro and in vivo*

**Figure 7:**
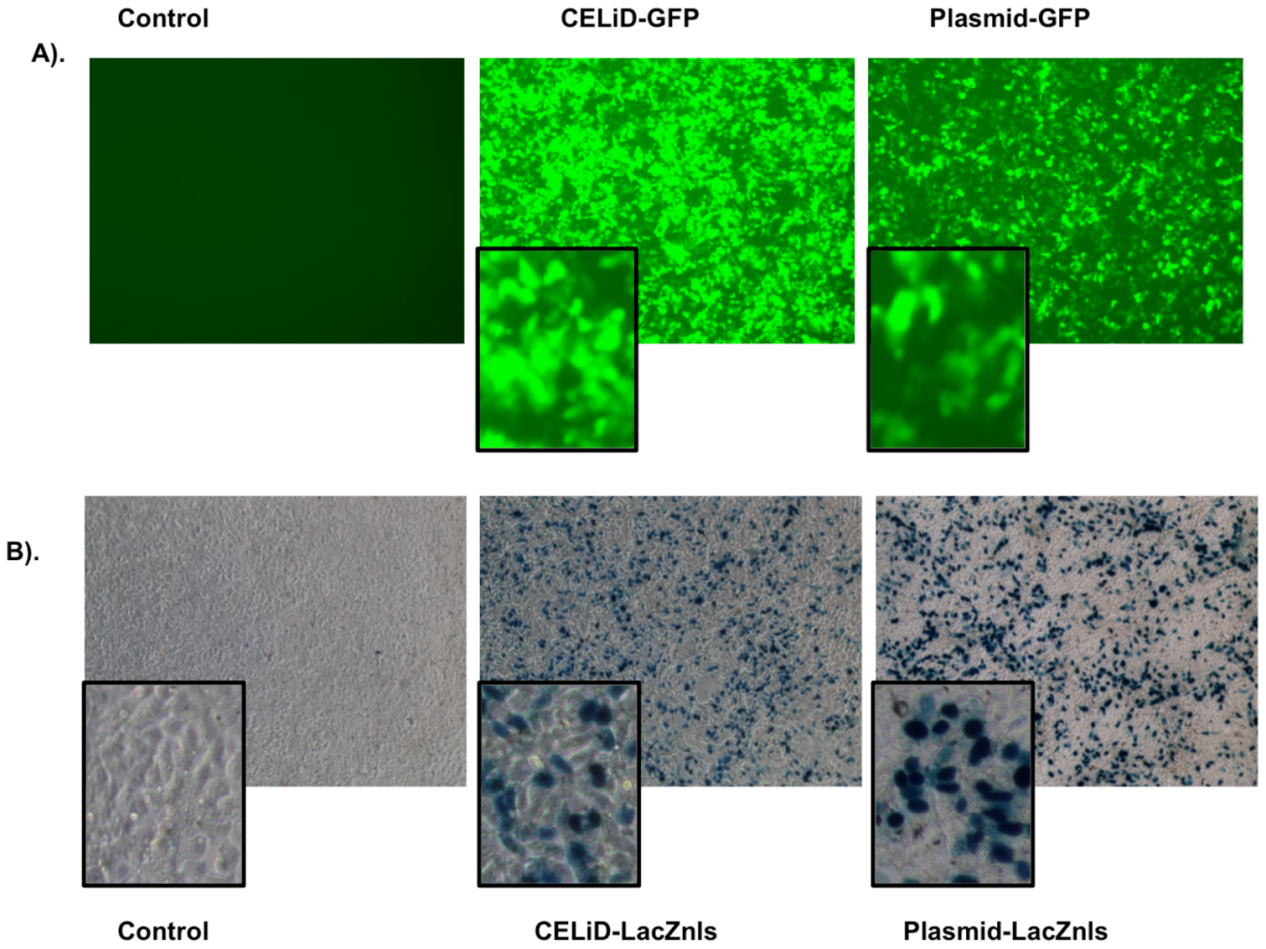
CELiD vector DNA expression *in vitro*. (A) HEK 293 cells transiently transfected with CELiD-GFP DNA or plasmid pFBGR for green fluorescent protein (GFP) expression. Both DNAs harbor an identical gene expression cassette encoding GFP and were transfected using equivalent copy numbers. Images were taken at day 6 post-transfection. Magnification, 10x objective (with digitally-enlarged insert). (B) HEK 293 cells transiently transfected with equal amounts of CELiD-LacZnls DNA or plasmid pFB-CMV-LacZnls. Both DNAs harbor an identical gene expression cassette encoding β-galactosidase with a nuclear localization signal (LacZnls). X-gal staining of cells at three days post-transfection. Magnification, 10x objective (with digitally-enlarged insert).

The ability of CELiD DNA produced in Sf9 cells to direct transgene expression *in vitro* was determined using an ITR-flanked green fluorescent protein reporter cassette (CELiD-GFP, Fig. 7A) or an ITR-flanked LacZnls (nuclear-localized β-galactosidase) reporter cassette (CELiD-LacZnls, Fig. 7B). Equivalent copy numbers of parental GFP plasmid and CELiD-GFP DNA molecules were used in the *in vitro* experiments. FACS analysis of cells transfected with CELiD-GFP or pFBGR (a GFP-expressing plasmid) showed that the population of positive cells and expression levels were similar: 39.6% and 31.1% GFP positive cells and 3656 and 2442 relative fluorescent units for CELiD and pFBGR-transfected cells, respectively (Fig. 7A). Using equal weight amounts of DNA, LacZ expression was higher for plasmid DNA than CELiD DNA in transiently transfected HEK 293 cells (Fig. 7B).

**Figure 8:**
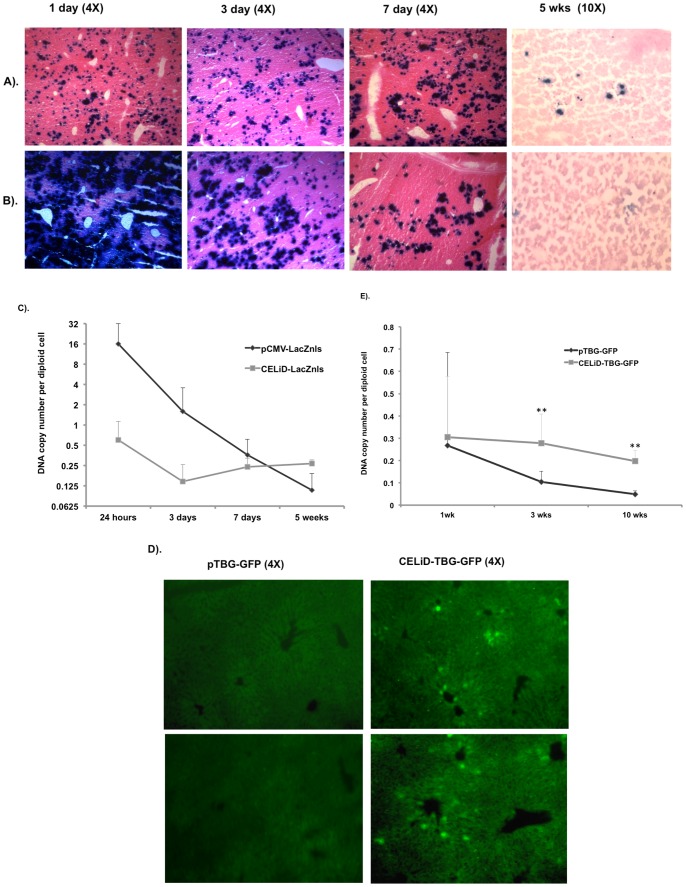
*In vivo* gene expression and DNA copy number per diploid cell following tail vein injection of (A) CELiD-LacZnls or (B) Plasmid-LacZnls (pCMV-LacZnls). 1 µg of CELiD DNA or 10 µg of the circular plasmid DNA was administrated by hydrodynamic tail vein injection. Transfected livers were harvested and processed at days 1, 3, and 7, as well as 5 weeks post-injection. Histological samples were sectioned and stained to detect β-galactosidase activity (indicated by dark blue nuclei). Samples were counterstained with eosin. (C) CELiD-LacZnls and plasmid-LacZnls copy number per diploid cell in transfected livers. The DNA copy number was normalized based on the PCR quantification of the endogenous mouse glucagon gene, n = 3 to 4. (D) Comparative long-term transgene expression from constructs bearing a liver specific thyroxine-binding globulin (TBG) promoter. CELiD-TBG-GFP or plasmid pTBG-GFP gene expression in liver section 10 weeks post-hydrodynamic tail vein injection. (E) CELiD-TBG-GFP and plasmid pTBG-GFP DNA copy number per diploid cell in transfected livers. The same amount of DNA (10 µg) was administrated by hydrodynamic injection. Statistical analysis by TTEST. ** indicates P<0.01.

*In vivo* gene expression was assessed in outbred ICR male mice using purified CELiD–LacZnls DNA administered by hydrodynamic tail vein injection (Fig. 8). Livers from mice injected with CELiD–LacZnls DNA (1 µg) had detectable levels of β-galactosidase activity at 1 day after injection, and the level of expression remained relatively constant through day 7. LacZnls activity could still be detected at 5 weeks post-injection in CELiD–LacZnls liver section (Fig. 8A). Plasmid pCMV-LacZnls DNA (10 µg) demonstrated robust expression at day 1, but quickly diminished over the course of the experiment. Although more plasmid DNA was injected than CELiD–LacZnls DNA, β-galactosidase activity in the pCMV-LacZnls-treated liver was not detectable at 5 weeks (Fig. 8B); however, the CELiD–LacZnls-treated liver still had detectable LacZnls expression at a similar time point. Quantitative PCR analysis of vector DNA copy number in transfected liver tissue showed that more CELiD-LacZnls DNA was retained in the liver at 5 weeks post-injection compared to plasmid DNA, and also showed a slower rate of decline in copy number over time (Fig. 8C). We postulate that the decline of LacZ expression over time is due primarily to shut-off of the CMV promoter in the liver. To address this hypothesis, a GFP reporter gene under the control of the thyroxine-binding globulin (TBG) promoter, which demonstrates prolonged activity in hepatocytes [14], [15], [16], was used to assess long-term gene expression in the mouse liver. CELiD-TBG-GFP and the equivalent plasmid pTBG-GFP DNA (10 µg each) were administrated to outbred ICR mice by hydrodynamic tail vein injection. At 10 weeks post-injection, no GFP expression was evident in the plasmid-treated mice, while GFP expression in livers of CELiD-TBG-GFP-treated mice was apparent (Fig. 8D). The CELiD-TBG-GFP expression pattern was predominantly centrilobular, resembling expression patterns observed in recombinant AAV-treated mouse livers [17]. Quantitative analysis of DNA copy numbers of CELiD-TBG-GFP and plasmid pTBG-GFP DNA showed very similar levels at 1 wk post-injection (Fig. 8E). CELID-TBG-GFP DNA copy number remained at 91% at 3 wks and 65% at 10 wks post-injection relative to the amount of vector DNA present at the 1wk time point, while plasmid pTBG-GFP DNA copy numbers decreased to 39% at 3 wks and only 1.8% at 10 wks post-injection relative to the 1 wk pDNA amount. Therefore, the amount of vector DNA in the liver of CELiD-TBG-GFP-recipient mice was 36-fold higher than those receiving equivalent plasmid DNA. The differences between CELiD-TBG-GFP and plasmid pTBG-GFP DNA copy numbers in transfected livers at 3 wks and 10 wks post-injection demonstrated statistical significance (P<0.01). The CELiD-TBG-GFP *in vivo* data revealed that the levels of CELiD DNA in mouse liver remained nearly unchanged during a 10 wk period.

## Discussion

Non-viral gene delivery circumvents certain disadvantages associated with viral transduction, particularly those due to the humoral and cellular immune responses to the viral structural proteins that form the vector particle [18], [19]and any *de novo* virus gene expression [2], [20]. Plasmids produced in *E. coli* contain elements needed for propagation in prokaryotes that are unnecessary, and even deleterious, for transgene expression in mammalian cells [21]. In addition to the required *cis*-elements, including a prokaryotic origin of DNA replication (e.g., colE1 ori) and a selectable marker, typically an antibiotic resistance gene (e.g., β-lactamase), bacterial plasmids bear uniquely prokaryotic modifications to DNA, such as N6-methyladenine and N5-methylcytosine [22]. These may elicit undesirable outcomes in an *in vivo* application [23]. Relative to mammalian genomic DNA, the occurrence of CpG dinucleotides in prokaryote-derived pDNA is overrepresented [24] and reportedly binds a member of the Toll-like family of receptors, eliciting a T cell-mediated immune response [25], [26], [27]. In addition, reducing the amounts of detrimental impurities, including bacterial genomic DNA and endotoxins, is critical for production of plasmids used *in vivo*.

“Mini-circle” DNA, a recent improvement to the production of pDNA free of prokaryotic *cis*-acting elements, uses an inducible recombinase to specifically delete the prokaryotic moiety from the bacterial mini-circle shuttle plasmid [28], [29]. Mini-circle DNAs have been used *in vivo* for long-term gene expression in mice, and for generating induced pluripotent stem cells (iPSC) *in vitro* [30]. Another alternative to pDNA is mini-linear, covalently closed (mini-lcc) DNA that possesses all the benefits of “mini-circle” DNA vectors and effectively eliminates the potential for undesirable vector integration events [31]. However, both mini-circle and mini-lcc DNA are produced in prokaryotes. We have described an alternative method for the high-yield production of eukaryote-derived, prokaryotic-sequence-free DNA for gene transfer. CELiD-vector DNA consists solely of a therapeutic or reporter gene transcription unit and the AAV2 ITRs. The *cis*-acting elements and *trans*-acting requirements needed for propagating the parental plasmid in *E. coli* are eliminated from the CELiD “rescue” and replication process. The genetic organization of CELiD-vector DNA, resembles rAAV vector DNA, but differs in conformation. The encapsidated recombinant AAV vector genome is predominantly a unique sequence of linear single-stranded DNA, although self-complementary forms are packaged either by designing a vector genome with a defective ITR [32], [33], [34], or through natural processes whereby half wild-type-length genomes are packaged as self-complementary DNA [35]. Unlike encapsidated AAV vector genomes, CELiD-vector DNA has no packaging constraints imposed by the limiting space within the viral capsid. In theory, the only size limitation resides in the DNA replication efficiency of the host cell. Infection of a stable cell line bearing an integrated copy of a 2.7 kb GFP vector DNA with a Rep-expressing baculovirus can yield up to 60 mg of CELiD-vector DNA from 4.7×10^9^cells. This quantity of CELiD DNA represents: 1) ≥4 million copies per cell, 2) approximately 15– times the mass of the cellular genomic DNA, and 3) approximately 0.36% of the cell mass. Thus, CELiD-vector DNA represents a viable eukaryotic alternative to prokaryote-produced pDNA.

### CELiD as a biological replicative intermediate

Autonomous, linear DNA replicons with covalently closed ends (i.e., terminal hairpins) have been identified in a wide variety of prokaryotic and eukaryotic organisms. Examples include certain large DNA viruses of eukaryotes (such as poxviruses and African swine fever virus), as well as various temperate phage of gram-negative bacteria (such as N15 and φKO2 of *Escherichia coli* and *Klebsiella oxytoca*, respectively). Additionally, linear plasmids and chromosomes bearing covalently closed ends have been described in *Agrobacterium tumefaciens* and various spirochete species of the genus *Borrelia*. Linear replicon-encoded *trans*-acting factors responsible for terminal hairpin formation have been identified and biochemically characterized. In particular, the ResT protein of *Borrelia* and the *TelN* protein of phage N15 have been extensively examined (reviewed in [36], [37]). We have demonstrated that a transgene sequence flanked by adeno-associated virus inverted terminal repeats (i.e., CELiD DNA) is maintained within *S. fugiperda* insect cells as a covalently closed, linear DNA molecule that serves as an AAV Rep protein-dependent replicon. Interestingly, the large AAV Rep proteins share biochemical activities with the ResT and TelN proteins, including DNA binding activity, tyrosine-mediated phosphodiesterase activity and the ability to join cleaved DNA strands [38], [39], [40], [41], [42]. We postulate that Rep-mediated DNA cleavage-joining activity is responsible for generation of the covalently closed DNA structure observed for CELiD DNA.

Perhaps the best-known examples of covalently closed linear replicons in eukaryotes are those of the *Poxviridae*. Poxvirus DNA replication bears similarities to that of the *Parvoviridae* (including the dependoviruses such as AAV). DNA replication in both virus families initiates by enzymatic nicking of one of the terminal hairpins flanking each end of the linear genome [43], [44]. The nicking event provides a 3′-hydroxyl group to prime strand-displacement replication. In addition to providing a self-priming DNA element to initiate DNA synthesis, covalently closed terminal hairpin structures may protect the linear DNA elements from attack by cellular exonucleases, as well as inhibit illegitimate recombination events. The role, if any, that closed-ended replicative forms play in wild-type AAV replication remains to be determined.

## Supporting Information

Figure S1 Restriction endonuclease digestion products resolved using native and alkaline agarose gel electrophoresis. (A) Native (1% agarose, TBE) and (B) denaturing (0.7% agarose, alkaline) gel electrophoresis. For both gels, CELiD-GFP DNA samples in lanes 1–6 were digested with the following restriction endonucleases. Lane 1: AgeI (monomer endonuclease recognition site is at position 1801, fragment sizes 1801/867 bp). Lane 2: SpeI (recognition site is at position 2456, fragment sizes 2456/212 bp). Lane 3: XhoI (recognize site is at position 493, fragment sizes 493/2175 bp). Lane 4: KpnI (recognize site is at position 410, fragment sizes 414/2254 bp). Lane 5: NotI (recognize sites at positions 195 and 2471, fragment sizes 195/2471/196 bp). Lane 6: XbaI (recognize sites for monomer at positions 188 and 2479, fragment sizes 188/2479/189 bp). Lane 7: undigested CELiD-GFP DNA. Monomer CELiD-GFP full-length size  = 2668 bp. (C) Table of predicted restriction endonuclease digestion products. AgeI, SpeI, XhoI and KpnI have single recognition sites, whereas NotI and XbaI have two recognition sites in the CELiD-GFP monomer sequence. Digestion products resolved using native agarose gel electrophoresis produced DNA fragments predicted by the rAAV-GFP (and corresponding CELiD) map, listed in the table by fragment length (x, y, or z) in basepairs. The denaturing, alkaline agarose gel products were 2x and 2y nucleotides in length for the single-cut enzymes, whereas the double-cut enzymes produced 2x and 2z length products, and an internal product y that appears the same size on both native and denaturing gels. Schematic representation of (D) single-cut and (E) double-cut restriction enzyme sites in monomer and dimer forms of CELiD vector DNA. The open boxes indicate the positions of the AAV ITRs. The horizontal arrow, collinear with CELiD vector DNA, indicates the direction of the upper, sense-strand in panels D and E. The two dimer figures represent tail-to-tail and head-to-head configurations. A tandem head-to-tail configuration would produce x+y fragments, which were not detected. (TIF)

Figure S2 Anti-Rep protein western blot of CELiD-GFP DNA samples treated with *Serratia marcescens* endonuclease. CELiD-GFP DNA samples were treated with 6.7 units of *S. marcescens* endonuclease (Turbonuclease, Accelegen, Inc., San Diego, CA, USA) (except for the material in lane 5, which was treated with 26.8 units) at 37°C for 30 min prior to SDS-polyacrylamide gel electrophoresis (SDS-PAGE) and western blotting. The quantities of CELiD-GFP DNA treated with endonuclease were as follows: Lane 1: 8 µg; lane 2: 16 µg; lane 3: 32 µg; lane 4: 64 µg; lane 5: 64 µg. After SDS-PAGE, proteins were electrophoretically transferred to a nitrocellulose membrane and probed with an anti-Rep monoclonal antibody as described in Materials and Methods, except that a 1∶2000 dilution of the secondary detection antibody was used. (TIF)
