# Supplementary figures and images for: Production and Characterization of Novel Recombinant Adeno-Associated Virus Replicative-Form Genomes: A Eukaryotic Source of DNA for Gene Transfer
Source: PLoS One. 2013 Aug 1;8(8):e69879. doi: 10.1371/journal.pone.0069879 (PMC3731302; doi:10.1371/journal.pone.0069879)

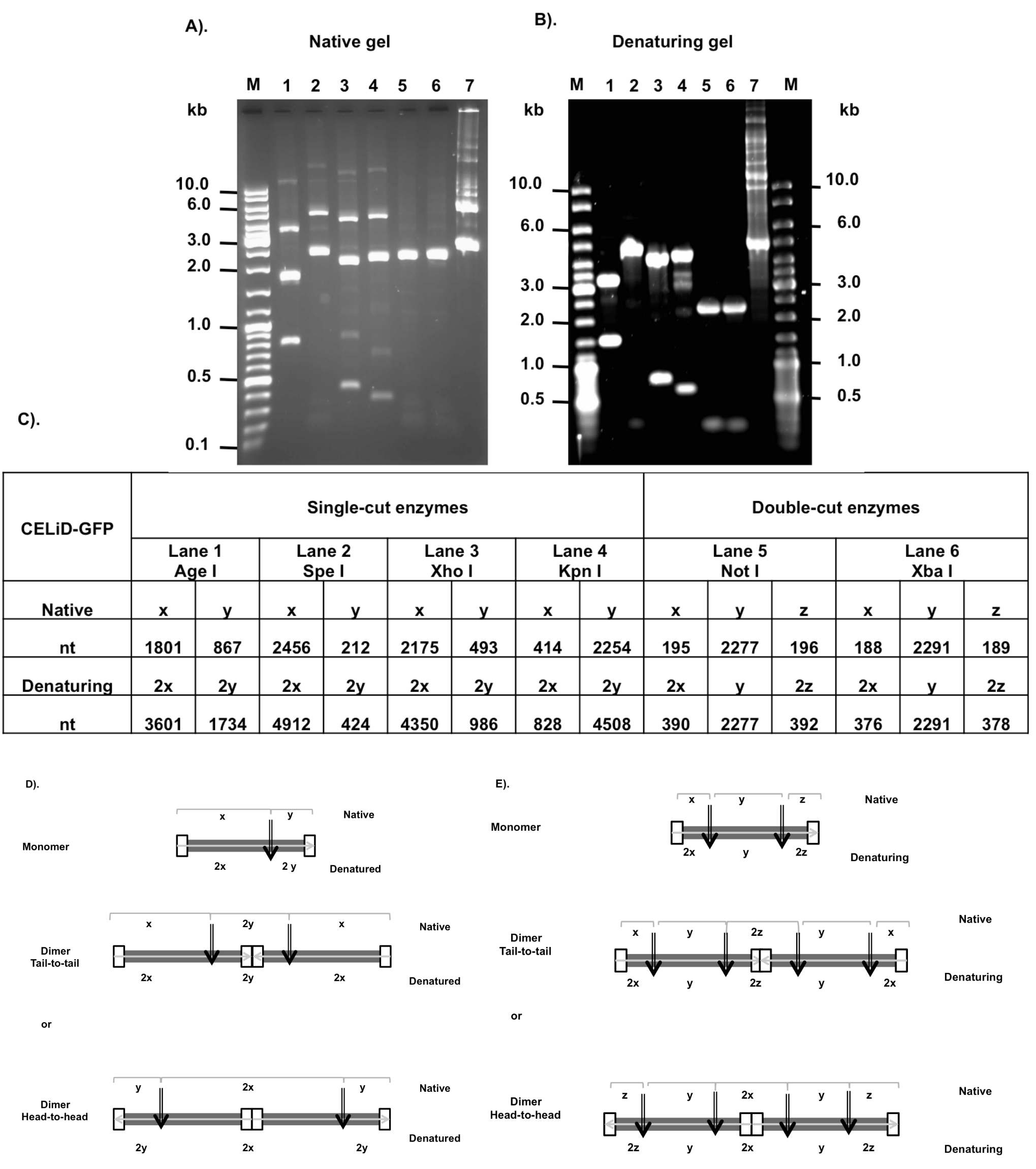

Supplement: Figure S1 — (TIF) [file pone.0069879.s001.tif]
